# Energy and structure of bonds in the interaction of organic anions with layered double hydroxide nanosheets: A molecular dynamics study

**DOI:** 10.1038/srep19986

**Published:** 2016-01-28

**Authors:** A.A. Tsukanov, S.G. Psakhie

**Affiliations:** 1 Skolkovo Institute of Science and Technologies, Moscow, 143026, Russia; 2 Tomsk Polytechnic University, Tomsk, 634050, Russia; 3 Institute of Strength Physics and Materials Science SB RAS, Tomsk, 634055, Russia

## Abstract

The application of hybrid and hierarchical nanomaterials based on layered hydroxides and oxyhydroxides of metals is a swiftly progressing field in biomedicine. Layered double hydroxides (LDH) possess a large specific surface area, significant surface electric charge and biocompatibility. Their physical and structural properties enable them to adsorb various kinds of anionic species and to transport them into cells. However, possible side effects resulting from the interaction of LDH with anions of the intercellular and intracellular medium need to be considered, since such interaction can potentially disrupt ion transport, signaling processes, apoptosis, nutrition and proliferation of living cells. In the present paper molecular dynamics is used to determine the energies of interaction of organic anions (aspartic acid, glutamic acid and bicarbonate) with a fragment of layered double hydroxide Mg/Al-LDH. The average number of hydrogen bonds between the anions and the hydroxide surface and characteristic binding configurations are determined. Possible effects of LDH on the cell resulting from binding of protein fragments and replacement of native intracellular anions with delivered anions are considered.

Naturally occurring hydroxide-based layered minerals, in particular anionic or cationic clays, have unique physical and chemical properties, owing to their layered structure, consisting of charged hydroxide layers that are “glued” together by charge-balancing counter-ions. Almost any kind of ion can serve as an electrostatic binder, meaning that both anionic and cationic clays are capable of hosting simple single-atomic inorganic ions^1^,^2^, as well as large charged organic species, such as genes^3^,^4^, amino acids^5^,^6^ and drug molecules^7^,^8^. This is one of the reasons why hybrid and hierarchical nano-objects based on layered hydroxides and oxyhydroxides are seen to hold great promise in the biomedical field^9^,^10^,^11^.

Besides the natural stacked host-guest structure, hierarchical core-shell nano-objects based on hydroxides and oxyhydroxides of metals are known^12^,^13^,^14^. Such particles consist of a metallic or partially metallic core surrounded by a shell of layered metal hydroxide or aluminum oxyhydroxide. Other shapes, such as hollow-shell and yolk-shell structures, have been synthesized as well^15^,^16^.

The presence of a large electric charge and a sufficient number of periodically distributed hydroxyl groups on both sides of each nanosheet of the layered hydroxide determines their ability to form a large variety of non-covalent bonds with many types of molecule. This allows layered hydroxides to be used as building blocks for the encapsulation, storage, transport and delivery of almost any type of nano-agent^17^,^18^,^19^. Layered hydroxide-based nanoparticles, in combination with ferromagnetic compounds, also allow for targeted delivery through an externally applied magnetic field^20^,^21^,^22^,^23^. Such magnetic nano-objects can further be used for the separation of proteins^24^ and for medical imaging^23^.

A single layer of anionic clay is a positively charged nanosheet of metal hydroxide that can be viewed as an independent object^25^,^26^. This quasi-two-dimensional nano-object can be obtained, for example, via LDH delamination^27^,^28^. The use of LDH-based nano-objects for biomedical applications may affect the ionic environment in the intercellular and intracellular medium, possibly influencing potential-dependent processes in the cell. In particular, when LDH-based nanoparticles are used as carriers/capsules for the delivery of drugs and genes, fragments of LDH nanosheets remain in the cell medium after delivery of the agent. These nanosheets of LDH generally have a significant surface charge density^29^ (the charge is assumed to be positive, and its density is determined by the number of trivalent metal atoms M^III^ per unit area of LDH). This allows LDHs to act as effective adsorbents or even “traps” for anions of the cell medium. Thus, the capture of anions of the extracellular and/or intracellular medium is one of the possible side-effects (not necessarily adverse) of hybrid and hierarchical LDH-based agents. To understand such side effects, it is desirable to understand the specifics of bonding between anions of the cell medium and fragments of LDH.

The most prevalent anions in the extracellular medium are the chloride Cl^−^ and bicarbonate HCO_3_^−^ ions. The intracellular medium, however, has a much more complex ionic composition. Inside the cell, negative charge is present in high-molecular-weight compounds, mostly protein molecules with negatively charged amino acids – glutamate and aspartate, and also in organic phosphates. Apart from this, the intracellular medium also contains bicarbonate and chloride ions.

The aim of the present work is to investigate the interaction of organic anions – aspartic amino acid anion, glutamic amino acid anion and bicarbonate ion (which occur free or as part of larger molecules and together represent the main anions of the cell) with a single nanosheet of Mg/Al-LDH.

## Results

### Molecular dynamics simulations

Computer simulations, especially molecular dynamics (MD) and MD-based methods form a powerful and flexible instrument for the investigation of complex molecular systems, including LDH and LDH-based nanohybrids^30^. MD methods are widely used to study the interaction of ions and water molecules with the surface of layered hydroxides and oxyhydroxides of metals^31^,^32^,^33^,^34^,^35^,^36^. The structure and properties of host-guest systems of LDH with intercalated inorganic and organic species were also studied using MD^2^,^37^,^38^,^39^,^40^,^41^,^42^,^43^,^44^,^45^,^46^. In particular, Newman *et al.* have investigated phenylalanine and tyrosine amino acids (in anionic and cationic states) inside the interlayer region of Mg/Al-LDH and a cationic clay montmorillonite^46^. The arrangement of water and amino acid molecules, possible hydrogen-bond interactions between guest-guest (amino acid – water) and host-guest were characterized^46^. Steered MD approach^47^ was utilized for study of such systems as well^48^. The host-guest complex of LDH and glutamic amino acid was studied experimentally in^49^.

Previous MD studies have shown that the surface of layered hydroxides forms hydrogen bonds with organic molecules, such as amino acids^46^, drugs^37^ and polymers^50^. It was also shown, both experimentally and employing MD simulations, that a single nanosheet of aluminum-based oxyhydroxide can bind anionic phospholipids and even capture a bacterial cell, attracting its lipid membrane^51^.

**Figure 1:**
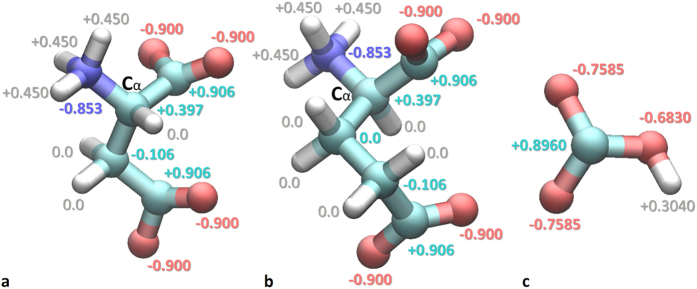
Partial atomic charges of polyatomic anions in the model: (**a**) aspartic acid anion Asp A^−^, (**b**) glutamic acid anion Glu A^−^, (**c**) bicarbonate ion HCO_3_^−^. Both Asp A^−^ and Glu A^−^ are in the zwitterionic state. Colors: red – oxygen, purple – nitrogen, cyan – carbon, white – hydrogen.

To quantify the free energy of adsorption and to understand the binding configurations of the organic ions with a fragment of Mg/Al-LDH, two series of MD simulations were performed: **#1**, adsorption from an aqueous salt solution of aspartic amino acid anions (Asp A^−^), glutamic amino acid anions (Glu A^−^), bicarbonate ions HCO_3_^−^ (hydrocarbonate) and chloride ions Cl^−^ by a pure nanosheet of quintinite n[Mg_4_Al_2_(OH)_12_^2+^]; **#2**, estimation of the free energy of adsorption on the hydroxide surface for each kind of anion. Full-atom MD models were built and employed in all calculations. The positive net charge of the nanosheet was neutralized by choosing the appropriate concentration of anions. The chosen concentrations of anions do not necessarily correspond to actual concentrations found inside cells. Partial atomic charges of the polyatomic anions used in the MD are shown in Fig. 1.

In **#1**, an MD simulation of the system in an explicit solvent was carried out. Typical locations and binding zones as well as the average amount of formed hydrogen bonds with the LDH surface were determined for each type of anion. In series **#2**, the free energy of adsorption of organic anions and chloride ions on the LDH surface was estimated using the steered molecular dynamics (SMD) approach^52^. Final configurations of adsorbed anions obtained in simulation **#1** were used as initial conditions for SMD simulations **#2**.

### H-bond formation between organic anions and LDH

**Figure 2:**
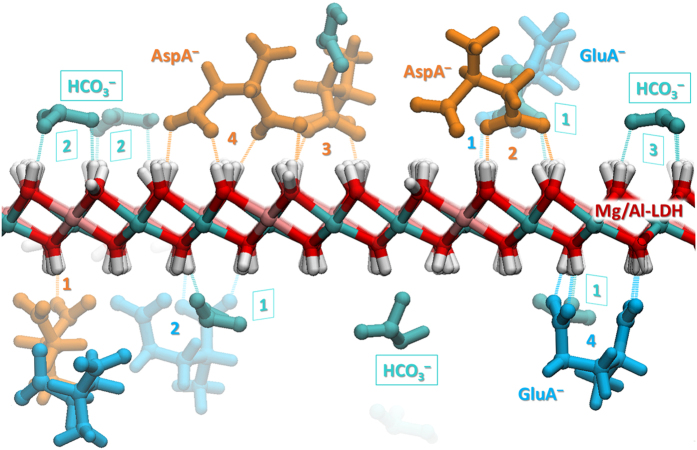
The formation of hydrogen bonds between the carboxyl groups of organic anions and the surface hydroxyl groups of a single nanolayer of LDH. The dotted lines represent H-bonds. The numbers beside molecules indicate the number of H-bonds formed. Ion color code: orange – Asp A^−^, blue – Glu A^−^, cyan – bicarbonate anions. LDH color code: red – oxygen, pink – aluminum, cyan – magnesium, white – hydrogen. Water and chloride are omitted for clarity.

The formation of hydrogen bonds (H-bonds) between the carboxyl oxygen atoms of the organic anions as acceptors and the hydroxyl groups of LDH as donors was observed. To estimate the number of H-bonds a geometric criterion was adopted. We assumed that a H-bond exists if the distance between donor and acceptor is less than 3.0 Å, at an angle 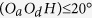. It is interesting to note that Asp A^−^, employing all its oxygen atoms for bond formation, frequently forms four H-bonds with LDH (more often than Glu A^−^) (Fig. 2). This may be explained by the difference in length of the hydrocarbon chains of the molecules. For the relatively long Glu A^−^ molecule, it is energetically more difficult to assume a fitting conformation, whereas Asp A^−^ fits well to the topology of OH-groups on the nanosheet surface. An example of this can be seen in Fig. 2 (bottom right), where a Glu A^−^ anion that has formed four H-bonds with LDH is strongly deformed along the C-Cα-…-C chain, forming a strained Π-like shape.

The bicarbonate ion also has suitable dimensions to form more than one bond with the layered hydroxide. The formation of 1 to 3 H-bonds per molecule was observed, three bonds being rare, however. This can be explained by two factors. Firstly, the bicarbonate anion is a much smaller and more rigid molecule, which may adversely affect the formation of multiple bonds per ion. And secondly, in our model, the partial charges on the oxygen atoms of Asp/Glu anions are slightly larger than in the HCO_3_^−^ molecule (see Fig. 1).

A part of the system at the final time step (5 ns) is depicted in Fig. 2. Hydrogen bonds are shown as dotted lines; the numbers indicate the number of bonds formed by the molecule. The average numbers 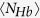 of H-bonds per anion (with the LDH surface) are listed in the Table 1. The largest number of H-bonds was observed for aspartic acid anions, the least for bicarbonate.

### Common binding sites of the anions with LDH

**Figure 3:**
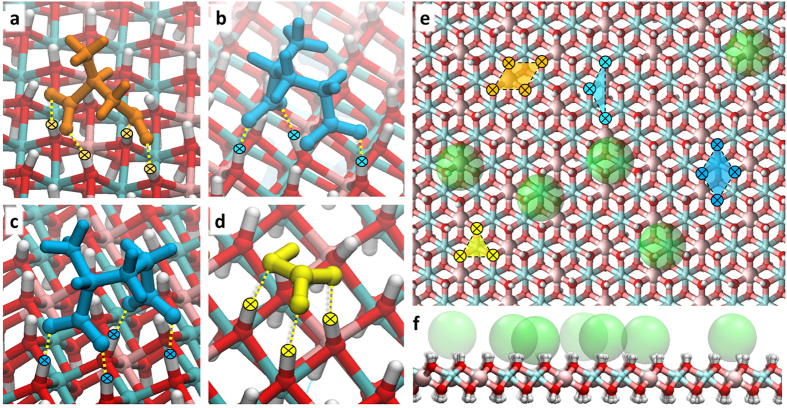
Common binding sites of anions with the LDH surface: (**a**) aspartic anion; (**b**,**c**) glutamic anions; (**d**) bicarbonate anion; (**e**) plane view of binding configuration on the LDH surface; (**f**) interaction of chlorine ions with the hydroxide. Ion colors: orange – Asp A^−^, blue – Glu A^−^, yellow – HCO_3_^−^, green – van der Waals spheres of chlorine; LDH colors are the same as in Fig. 2; yellow dotted lines – H-bonds.

Typical binding sites at the hydroxide surface and residence zones of organic and chloride anions are shown in Fig. 3. An aspartic acid anion forming four H-bonds with neighboring OH-groups of LDH is shown in Fig. 3a. The H-binding of a glutamic anion with the hydroxide surface in cases of three and four bonds formed is shown in Fig. 3b,c, respectively. In all these cases the binding points form obtuse triangles and rhombuses on the LDH surface, as can be seen in Fig. 3e (orange and blue). The three H-bonds between bicarbonate anion and the hydroxyl groups (Fig. 3d) form an equilateral triangle on the surface of the nanosheet (Fig. 3e, yellow). It can be also seen that chloride anions preferentially reside over the metal atoms, forming a tetrahedron with three adjacent hydrogen atoms of LDH (Fig. 3e).

### Free energy of adsorption of anions on the LDH surface

Interaction energies of organic anions and chloride with the Mg/Al-LDH nanosheet were estimated in terms of free energy of adsorption. Free energy estimates were obtained via calculation of the potential of mean force (PMF), which corresponds to the work of an external pulling force needed to remove an anion from its initial adsorbed state to the bulk water solution. To evaluate PMF profiles constant velocity steered MD^52^ simulations were performed for each anion type considered. In the case of polyatomic anions the external force was attached to a carbon atom, i.e. the central atom in HCO_3_^−^ or the Cα in amino acid anions (Fig. 1).

The SMD simulations were conducted in the complete system, including water and all anions, some of which were adsorbed on the surface. Therefore the obtained results do not represent the pure adsorption energy of a certain ion on the certain substrate, but the more realistic case where anions interact with each other, in particular, are linked together, forming H-bonds, replace each other occupying binding sites on the LDH surface, and screen electrostatic field. This probably approximates soft matter systems more realistically.

**Figure 4:**
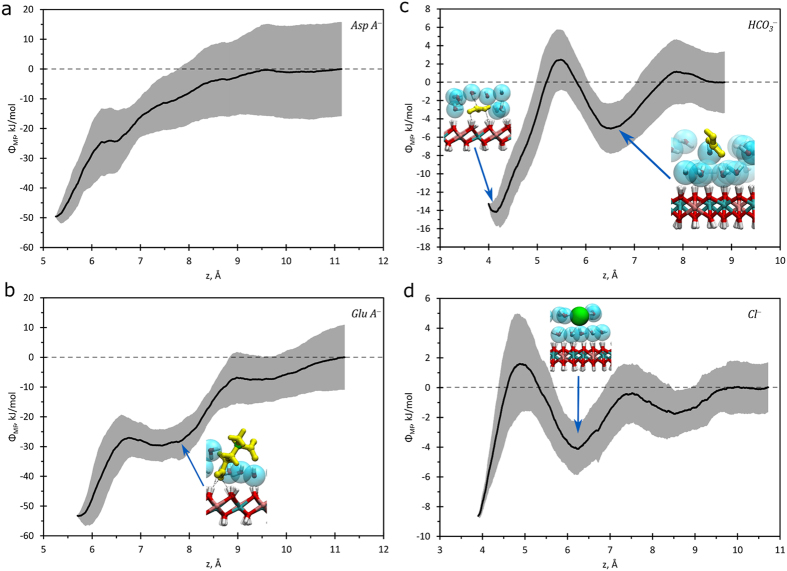
PMF profiles Φ(z) of adsorption of organic anions and chloride as a function of the distance between the reference atom and the center of the nanosheet. (**a**) aspartic anion; (**b**) glutamic anion; (**c**) bicarbonate anion; (**d**) chloride anion. Characteristic configurations of the anions and surrounding environment found at various points along the PMF profile are shown in the inserts.

Using the procedure described above, PMF profiles Φ(*z*) for each of the four anion types were obtained (Fig. 4). The PMF profile of the glutamic acid anion features a characteristic plateau (Fig. 4b). The first rise corresponds to breaking of H-bonds between the carboxyl group (the one closest to Cα) and LDH. The plateau itself corresponds to an energy-invariant rotation around the remaining H-bonds of the second carboxyl group. Similar behavior was found in the case of aspartic acid anion, although this cannot be seen on the averaged profile in Fig. 4a. The PMF profiles of bicarbonate (Fig. 4c) and chloride (Fig. 4d) anions are very similar and feature secondary local energy minima. This is due to the fact that both anions have dimensions comparable with a water molecule, and therefore have stable positions at distances corresponding to one or two mono-molecular layers of water from the surface.

Comparing of the total work required for removing the anions from the surface, it was found that the amino acid anions have much larger energies of adsorption than either bicarbonate or chloride anions (Table 1). This implies that the amino acid anions will bind to the LDH surface preferentially and displace chloride and bicarbonate ions. Also, LDH nanosheets will likely bind to protein fragments containing Asp/Glu amino acids, which may affect various cellular processes. The anion exchange at the surface of Mg/Al-LDH may disturb the ionic balance of the cellular medium, possibly affecting transport and signal processes, as well as nutrition and proliferation of the cell.

### Formation of multi-molecular complexes on LDH

**Figure 5:**
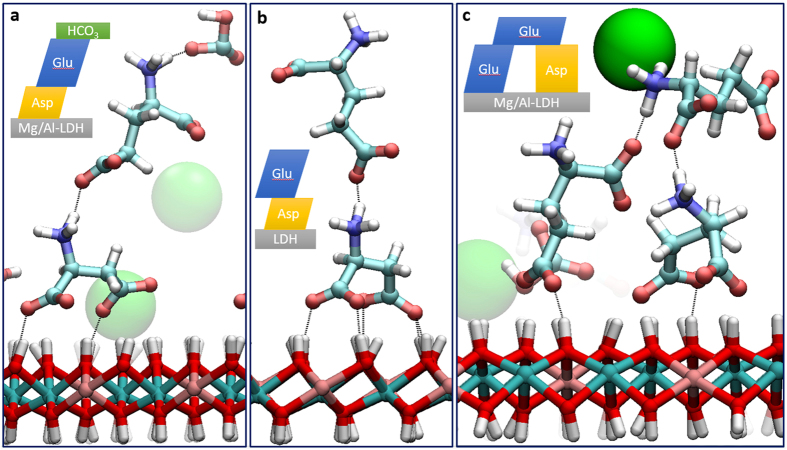
Snapshots of multi-molecular complexes on the LDH surface close to the final time step of simulation #1. (**a**) Example of a three-molecular linear system. (**b**) A two-molecular linear system. (**c**) A three-molecular horseshoe-like system, which forms a cycle of LDH-A_1_^−^-A_2_^−^-A_3_^−^-LDH. Color codes: anions – red = O, purple = N, cyan = C, white = H, green = Cl; LDH colors are the same as in Fig. 2. H-bonds are represented by dotted black lines. Water is not shown.

The MD simulation has also shown that anions of the two amino acids bind to the surface of LDH in such a fashion, that their positively charged amino groups are oriented away from the hydroxide and are accessible from the aqueous environment (Fig. 5). These sites of concentrated positive charge are open to the formation of electrostatic, hydrogen and even covalent bonds, e.g. peptide bonds with surrounding amino acid residues. This opens the possibility of using an LDH nanosheet to fix certain anions in a predictable orientation, and with high density, which may be useful for the synthesis of more complex hybrid or hierarchical nano-objects, or even protein fragments. The effect may even facilitate crystallization of proteins for structural studies. The results reported here can be expected to apply in a similar fashion to cationic clays, which would interact with positively charged organic ions.

## Discussion

The free energy of adsorption of organic anions Asp A^−^, Glu A^−^ and HCO_3_^−^ on the surface of Mg/Al-LDH were estimated using SMD simulations. It was found that aspartic and glutamic amino acid anions in the zwitterionic state strongly interact with the LDH surface, forming multiple hydrogen bonds. This is due to the specific “layout” and orientational mobility of the LDH hydroxyl groups as well as the flexibility of the organic anion molecules. It can be speculated that LDH will also bind to intracellular and membrane proteins that contain these amino acids.

Characteristic anion binding sites were identified on the LDH surface. The obtained results show that the “fine-structure” of charge on the LDH surface has a significant effect on the affinity of particular polyatomic anions to bind to the surface. Therefore, in MD studies of LDH-based nano-objects it is important to consider the all-atom structure of the nanomaterial, especially in cases of organic anion capture, interaction with protein fragments, lipid systems and other compounds with non-uniform electric charge distribution.

The MD simulation has also shown that, partly due to the zwitterionic form of the considered anions, multi-molecular complexes are formed on the surface of LDH, which are held together by H-bonds. The probability of formation of such complexes on the surface of LDH is higher than in bulk solution, due to the higher concentration of anions close to the surface, and a preferential orientation (amino groups pointing away from the surface). It seems possible that due to this property, LDH can be used like a “breadboard” (as in electronic circuits) for the synthesis of certain polypeptides or as a precursor for hybrid nano-structures.

In conclusion, it was shown that the free energies of adsorption on Mg/Al-LDH nanonsheets of Asp A^−^ and Glu A^−^ are significantly higher than those of Cl^−^ and HCO_3_^−^. Based on the obtained data it is possible to conclude that especially the amino acid anions will interact with LDH in terms of selective ion capture and anion exchange. It can be expected that aspartic and glutamic acid anions will replace chlorine and bicarbonate ions on the surface of the nanomaterial. In case of LDH intercalated with chloride anions, this effect can result in depletion of charged amino acids in the cell medium and the simultaneous release of large amounts of chloride ions into the intracellular medium, possibly disrupting cellular processes or even resulting in cell death.

## Methods

### Molecular dynamic model

To describe the Mg/Al-LDH nanosheet structure, X-ray crystallographic data from ref.^53^ was utilized. The CLAYFF force field^54^ was used for model parameterization. The coordinates of non-hydrogen atoms were frozen during the entire simulation. The models of Asp A^−^ and Glu A^−^ anions were built on the basis of the CHARMM^55^ force field, using SwissParam^56^. In addition, Asp A^−^ and Glu A^−^ were in the zwitterionic state (Fig. 1a,b). The bicarbonate HCO_3_^−^ ion model was made utilizing SwissParam as well, but partial atomic charges were obtained from an additional calculation with the Hartree-Fock method HF/6-31G**, ref.^57^ (Fig. 1с). Because the CLAYFF force field does not define Lennard-Jones (LJ) parameters for hydrogen, the CHARMM parameters r_0_ = 0.449 Å, ε = 0.046 kcal/mol^54^ were used. This modification does not influence the subsystem described by CLAYFF, because the LJ and Coulomb pairwise interactions are not considered for directly bonded atom pairs, or atoms separated by less than 3 covalent bonds.

The simulation box dimensions were 79.5 × 64.2 × 200.0 Å. Periodic boundary conditions were applied in all directions. The area of the LDH fragment was about 50 nm^2^, the nanosheet had the bruto formula 105[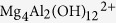], containing 210 aluminum atoms, which provide the LDH fragment with a positive net charge of 210 *e*. Initially, the ions were uniformly distributed in the simulation box: 70 Cl^−^, 70 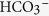, 35 Asp A^−^ and 35 Glu A^−^ anions. The system was first equilibrated under NVT conditions for 2 ns. After that, simulation **#1** was run for 15 ns. Molecular trajectories of the last 10 ns were utilized for analysis. The time dependence of the number of adsorbed anions for each anion type during this period is shown in supplementary Fig. S1. Final configurations of simulation **#1** were utilized as initial conditions in constant velocity steered MD simulations **#2**. The pulling velocity was *v* = 0.1 nm/ns, and the spring constant *k* = 1000 kcal/(mol·Å^2^). In case of amino acid anions the pulling force was attached to the alpha-carbon atom (Cα). Bicarbonate anions were pulled by applying the force to the central (carbon) atom. The duration of each SMD simulation was approximately 5 ns.

Parameters for Cl^−^ ions and TIP3P water were used in accordance with CHARMM27. The total number of atoms was about 100 000. All simulations were performed under NP_z_T conditions, at human body temperature (310 K) and 1 atm pressure. An integration time step of 1 fs was chosen.

## Additional Information

**How to cite this article**: Tsukanov, A.A. and Psakhie, S.G. Energy and structure of bonds in the interaction of organic anions with layered double hydroxide nanosheets: A molecular dynamics study. *Sci. Rep.* **6**, 19986; doi: 10.1038/srep19986 (2016).

## Supplementary Material

Supplementary Information

## Figures and Tables

**Table 1:** Average number of H-bonds formed with LDH per adsorbed organic anion (simulation **#1**) and free energy of anion adsorption on the Mg/Al-LDH surface (SMD simulations **#2**).

Anion	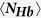	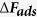 , kJ·mol^−1^
Aspartic acid A^−^	2.48 ± 0.35	49.6 ± 15.8
Glutamic acid A^−^	1.98 ± 0.26	53.3 ± 10.9
HCO_3_^−^	1.28 ± 0.09	13.3 ± 3.4
Cl^−^	–	8.7 ± 1.8
