# Supplementary material for: Energy and structure of bonds in the interaction of organic anions with layered double hydroxide nanosheets: A molecular dynamics study
Source: Sci Rep. 2016 Jan 28;6:19986. doi: 10.1038/srep19986 (PMC4730197; doi:10.1038/srep19986)
Supplement: Supplementary Information [file srep19986-s1.pdf]

Supplementary Information: Energy and structure of bonds in the interaction of organic anions with layered double hydroxide nanosheets: A molecular dynamics study

Alexey A. Tsukanov, Sergey G. Psakhie

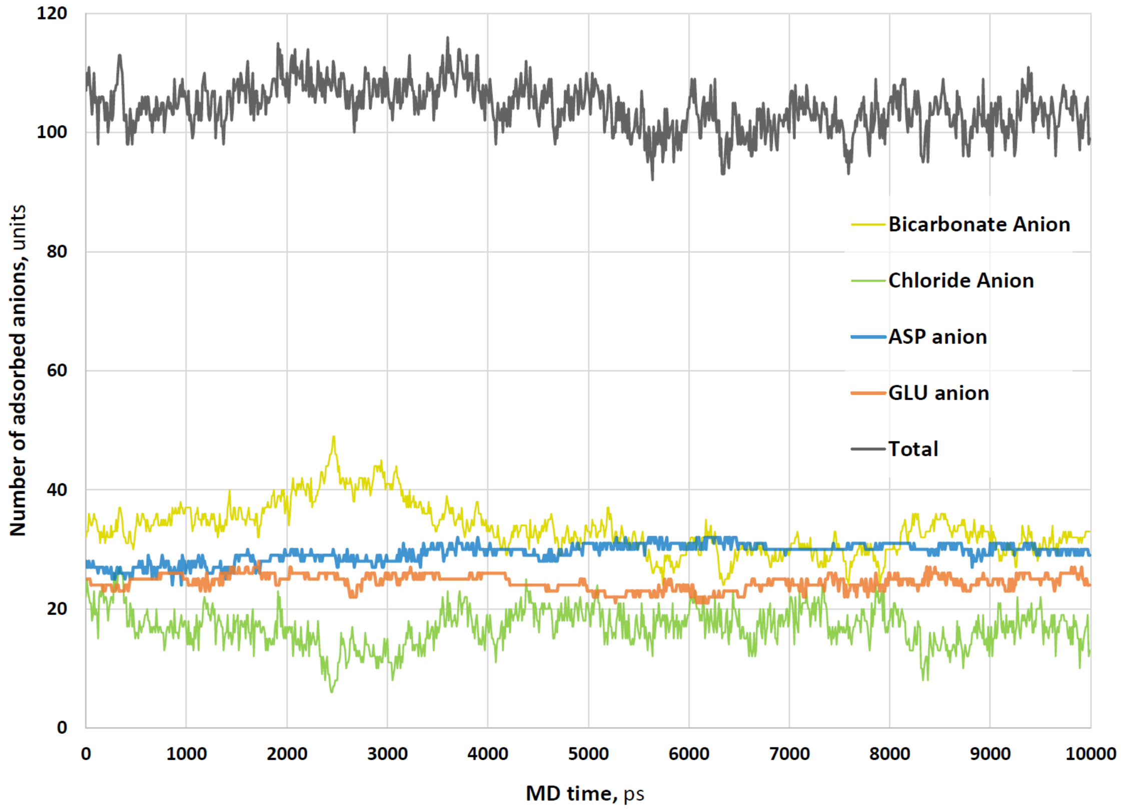

Figure S1: **Number of adsorbed ions for each anion type during the last 10 ns of MD simulation** (analysis period). Despite fluctuations (especially with bicarbonate and chloride), there is no discernible trend in the relative number of adsorbed anions, e.g. the modelled system is near equilibrium.
